# Screening Computed Tomography Angiography to Identify Patients at Low Risk for Delayed Cerebral Ischemia Following Aneurysmal Subarachnoid Hemorrhage

**DOI:** 10.3389/fneur.2021.740241

**Published:** 2021-11-12

**Authors:** Andrew M. Nguyen, Craig A. Williamson, Aditya S. Pandey, Kyle M. Sheehan, Venkatakrishna Rajajee

**Affiliations:** ^1^Department of Neurology, University of Michigan, Ann Arbor, MI, United States; ^2^Department of Neurosurgery, University of Michigan, Ann Arbor, MI, United States

**Keywords:** subarachnoid hemorrhage, cerebral vasospasm, computed tomography angiography, cerebral ischemia, intracranial aneurysm

## Abstract

**Introduction:** Delayed cerebral ischemia (DCI) occurs during a risk period of 3–21 days following aneurysmal subarachnoid hemorrhage (aSAH) and is associated with worse outcomes. The identification of patients at low risk for DCI might permit triage to less intense monitoring and management. While large-vessel vasospasm (LVV) is a distinct clinical entity from DCI, the presence of moderate-to-severe LVV is associated with a higher risk of DCI. Our hypothesis was that the absence of moderate-to-severe LVV on screening computed tomographic angiography (CTA) performed within the first few days of the DCI risk period will accurately identify patients at low risk for subsequent DCI.

**Methods:** This was a retrospective cohort study. Our institutional SAH outcomes registry was queried for all aSAH patients admitted in 2016–2019 who underwent screening CTA brain between days 4 and 8 following ictus. We excluded patients diagnosed with DCI prior to the first CTA performed during this time period. All variables are prospectively entered into the registry, and outcomes including DCI and LVV are prospectively adjudicated. We evaluated the predictive value and accuracy of moderate-to-severe LVV on CTA performed 4–8 days following ictus for the prediction of subsequent DCI.

**Results:** A total of 243 aSAH patients were admitted during the study timeframe. Of the 54 patients meeting the eligibility criteria, 11 (20%) had moderate-to-severe LVV on the screening CTA study performed during the risk period. Seven of the 11 (64%) patients with moderate-to-severe LVV on the days 4–8 screening CTA vs. six of 43 (14%) patients without, subsequently developed DCI. On multivariate analysis, the presence of LVV on days 4–8 screening CTA was an independent predictor of DCI (odds ratio 10.26, 95% CI 1.69–62.24, *p* = 0.011). NPV for the subsequent development of DCI was 86% (95% CI 77–92%). Sensitivity was 54% (25–81%), specificity 90% (77–97%), and positive predictive value 64% (38–83%).

**Conclusions:** The presence of moderate-to-severe LVV on screening CTA performed between days 4 and 8 following aSAH was an independent predictor of DCI, but achieved only moderate diagnostic accuracy, with NPV 86% and sensitivity 54%. Complementary risk-stratification strategies are likely necessary.

## Introduction

While mortality from aneurysmal subarachnoid hemorrhage (aSAH) has improved over the past few decades, morbidity remains high (1). Delayed cerebral ischemia, defined as the development of otherwise unexplained neurologic deterioration during the appropriate time period or radiographic evidence of cerebral infarction, is an important cause of poor outcomes following aSAH (1, 2). DCI typically occurs during a risk period of 3–21 days following aSAH. Up to 30% of aSAH patients may suffer DCI (3). While large-vessel vasospasm (LVV) was once considered the sole cause, DCI is now thought to be a complex entity, with inflammation playing a major role in the pathophysiology (2, 3).

While LVV may not be the sole cause of DCI, and in itself does not predict long-term outcomes, a strong correlation does exist between LVV and DCI. Several studies suggest that while DCI may occur in vascular territories without evidence of moderate-to-severe LVV, it is relatively uncommon in patients without significant LVV anywhere. This association between LVV and DCI is often leveraged to stratify DCI risk in asymptomatic patients, with patients at high risk receiving more intense monitoring in the Intensive Care Unit (ICU), and those at lower risk, without other indications for ICU care, potentially managed in lower intensity settings, such as moderate care (3). Such risk stratification may permit more cost-effective care and optimize ICU capacity for critically-ill patients. Some centers therefore perform screening CTA between days 4 and 8 to risk-stratify aSAH patients. While this practice is popular at several centers, and CTA is highly accurate for the detection of vasospasm outside the distal vasculature (4), the value of CTA performed on asymptomatic patients during the 4–8-day period has not been studied.

Our objective therefore was to evaluate predictive value and diagnostic accuracy of CTA performed on asymptomatic patients during the 4–8-day period to identify patients at low subsequent risk of DCI.

## Materials and Methods

This was a retrospective observational study, using prospectively collected data from a single center disease-specific registry. The Institutional Review Board (IRB) determined that this study is exempt from IRB regulation (HUM00037496). All adult (age 18 or older) patients with angiographically-confirmed aSAH admitted between January 2016 and June 2019 who underwent CTA 4–8 days following ictus were included. We excluded patients with a clinical diagnosis of DCI prior to the first CTA performed during this period. The data source was the University of Michigan Subarachnoid Hemorrhage (SAH) outcomes database (UMSAHOD). All patients with SAH (aSAH and angiogram-negative SAH) admitted to the University of Michigan are entered into this database. All data are prospectively entered, including demographics, comorbidities, baseline clinical variables including clinical grade, radiological findings, grade on admission, aneurysm location, treatment method, in-hospital events/complications, and outcomes. In-hospital events such as DCI and LVV are prospectively adjudicated in monthly or bi-monthly meetings of neurocritical care faculty investigators, at which at least two faculty members are present to adjudicate key variables including LVV as well as outcomes such as DCI. Documentation of DCI in the UMSAHOD is based on a neurocritical care faculty investigators' review of the entire medical record for the admission, for the presence of otherwise unexplained clinical deterioration during the appropriate period of risk (days 3–21 following ictus) or the appearance of delayed infarction on imaging. Documentation of moderate or severe LVV in the UMSAHOD is based on subjective review of the CTA by neurocritical care faculty investigators. While CONSCIOUS-1 criteria are broadly applied based on a subjective estimate of reduction in vessel caliber, direct measurement with calipers is not routinely performed (5). The presence of moderate-to-severe vasospasm in any one of the following segments was necessary to diagnose LVV: intracranial internal carotid artery, middle cerebral artery M1 or M2 segments, anterior cerebral artery A1 or A2 segments, posterior cerebral artery P1 or P2 segments, basilar artery, or intracranial vertebral artery.

### Management of Aneurysmal Subarachnoid Hemorrhage and Delayed Cerebral Ischemia

Following the diagnosis of aSAH, every attempt was made to secure the aneurysm *via* endovascular coiling or microsurgical clipping within 24 h of hospital admission. All patients were admitted to the neurointensive care unit. Nimodipine was administered for 21 days. Transcranial Doppler evaluation was performed daily for 14 days, starting at the day of admission. Fluid administration was targeted to euvolemia. Neurological decline attributable to DCI was typically evaluated using a combination of CTA, CT perfusion (CTP), and digital subtraction angiography. In the absence of clinical decline, performance of screening CTA for risk-stratification was at the discretion of the attending neurointensivist or neurosurgeon. Suspected DCI was treated with a combination of hemodynamic augmentation in all cases, and endovascular therapy in some patients with moderate-to-severe LVV.

### Statistical Analysis

Descriptive analysis was performed using proportion and percentage for categorical variables, and median with interquartile range for continuous variables. Associations between categorical variables and outcomes of interest were tested for statistical significance using the Chi-square or Fisher exact test as appropriate. Associations between continuous variables and outcomes of interest were tested for statistical significance using the Mann–Whitney *U*-test. In order to study the predictive value of screening CTA, we performed multivariate analysis using binary logistic regression with the occurrence of DCI as the dependent variable. Covariates in the logistic regression model were selected based on prior evidence of association with DCI and biological plausibility (6). These included age, gender, Hunt, and Hess grade, modified Fisher grade, treatment modality (clipping, coiling, or neither), and the presence of LVV on day 4–8 screening CTA. The diagnostic accuracy of days 4–8 screening CTA to identify patients who develop DCI was studied, including sensitivity, specificity, negative predictive value (NPV), and positive predictive value (PPV). Diagnostic test positivity was defined as the prospective determination of the presence of moderate or severe LVV on CTA performed during this period by a neurointensivist as documented in the UMSAHOD. The gold standard (disease positivity) was the prospective determination of the occurrence of DCI following screening CTA based on review of all admission records as documented prospectively in the UMSAHOD.

## Results

**Figure 1:**
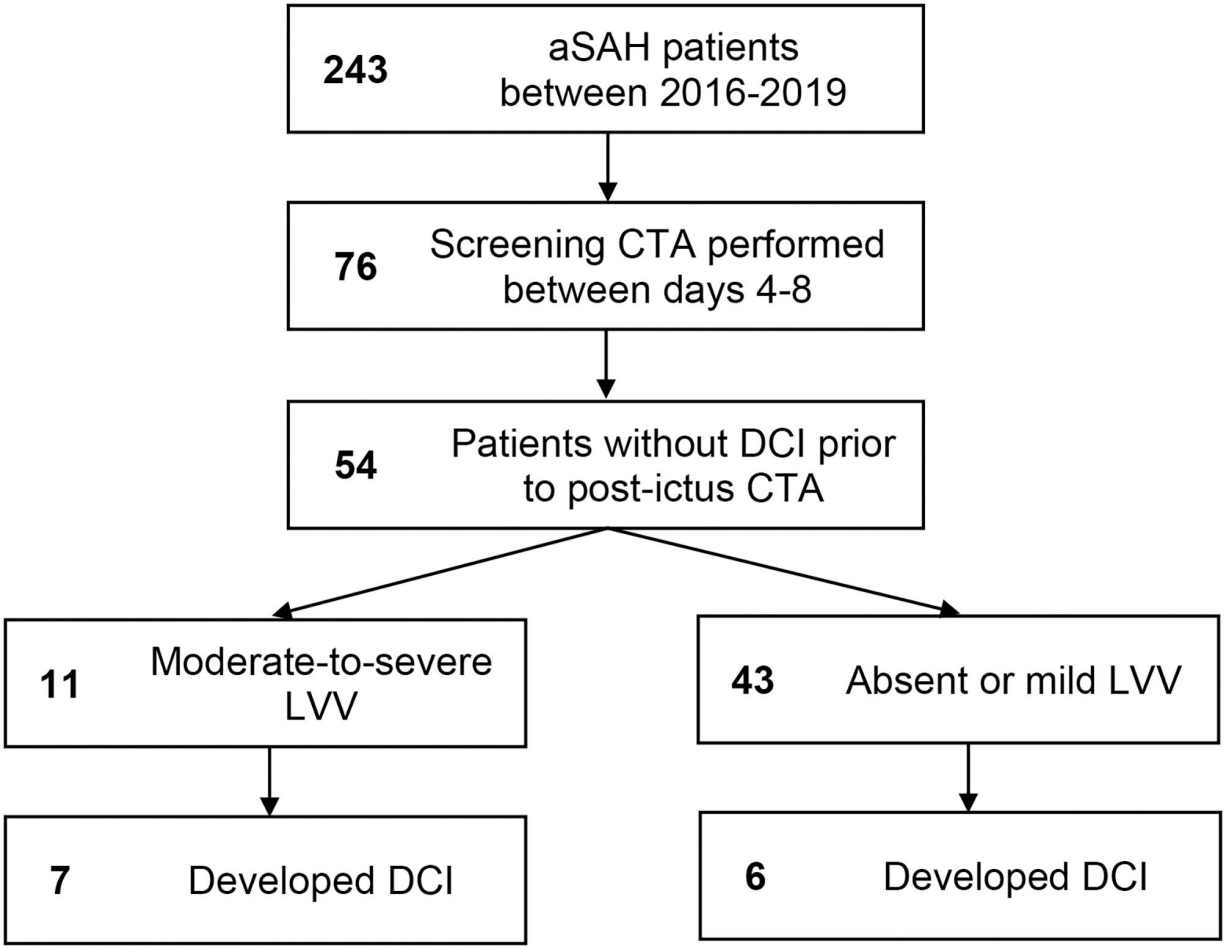
Flowchart of patients enrolled in the study.

A total of 243 aSAH patients were admitted during the study period (2016–2019). Of these, 76 (31%) underwent CTA during the 4–8-day window following ictus. Of these, 22 were excluded for occurrence of DCI prior to the CTA study. Patient baseline characteristics are in Table 1. Patient selection flow is outlined in Figure 1. Of the 54 patients meeting eligibility criteria, 11 (20%) had moderate-to-severe LVV on a screening CTA study performed during the risk period, and a total of 13 (24%) developed DCI. Seven of 11 (64%) patients with moderate-to-severe LVV on the days 4–8 screening CTA vs. six of 43 (14%) patients without, subsequently developed DCI (*p* = 0.002). On multivariate analysis, the presence of LVV on days 4–8 screening CTA was an independent predictor of DCI (odds ratio 10.26, 95% CI 1.69–62.24, *p* = 0.011). No other variable attained statistical significance once LVV was added to the model. The NPV of CTA performed during days 4–8 for the subsequent development of DCI was 86% (95% CI 77–92%). Sensitivity was 54% (25–81%), specificity 90% (77–97%), and PPV 64% (38–83%).

**Table 1:** Patient baseline characteristics.

**Variable**	**All subjects *N* = 54**	**DCI absent *N* = 41**	**DCI present *N* = 13**	***P*-value (univariate)**
Age in years, median (IQR)	62 (53–73)	63 (53–73)	56 (45–69)	0.15
Gender - Female	41 (76%)	30 (73%)	11 (85%)	0.40
Hunt and Hess grade, median (IQR)	3 (2–4)	3 (2–4)	4 (2–4)	0.16
Modified Fisher grade, median (IQR)	3 (3–3)	3 (3–3)	3 (3–3)	0.21
Treatment modality -Clipping -Coiling -Neither	15 (28%) 31 (57%) 8 (15%)	11 (27%) 23 (56%) 7 (17%)	4 (31%) 8 (62%) 1 (7%)	0.86
LVV on CTA	11 (20%)	4 (10%)	7 (54%)	0.001

*DCI, delayed cerebral ischemia*.

*IQR, interquartile range*.

*LVV, large-vessel vasospasm*.

*CTA, computed tomographic angiography*.

## Discussion

In our study, the presence of LVV on screening CTA performed on days 4–8 following aSAH was an independent predictor of DCI, but attained only moderate diagnostic accuracy in this role. While the NPV was 86%, sensitivity was only 54%. Therefore, based on the findings of our study, while only 14% of patients with mild or no LVV on days 4–8 screening CTA will develop DCI, about 4–5 of every 10 patients who subsequently develop DCI will be misclassified. These findings suggest that while screening CTA may be a useful tool for risk stratification, it cannot be relied upon in isolation. These findings are important because the use of screening CTA for risk-stratification during the DCI risk period is quite common in clinical practice (3). An important rationale for this practice is that the identification of aSAH patients at low risk for DCI may permit more targeted utilization of resources, especially ICU capacity. It has been estimated in one region that the length of stay for SAH patients may be $4,400 per day (7).

Our study suggests that while LVV and DCI are closely related, this widely utilized strategy focused on the detection of LVV will miss a potentially clinically relevant minority of aSAH patients who develop DCI. Several studies have examined the relationship between LVV and DCI. A *post-hoc* analysis of the CONSCIOUS-1 trial revealed that cerebral infarction rarely occurs (3%) in patients with mild or no LVV, while the presence of LVV was a strong independent predictor of infarction (8). In one study of aSAH patients evaluated with CTA and CTP, the presence of severe LVV was associated with a significant reduction in perfusion in the corresponding vascular territory, although this association was not as strong for moderate vasospasm (9). The flow territory that was least perfused corresponded to the vessel with the most severe vasospasm in only 65% of patients with moderate-to-severe LVV. A study that evaluated 25 aSAH patients with the gold standard diagnostic tools of DSA for LVV and ^15^O Positron Emission Tomography (PET) for ischemia found that the cerebral blood flow was lower and oxygen extraction fraction higher in brain regions supplied by vessels with significant LVV. However, hypoperfusion was also seen in 24% of patients without LVV (10).

A study that examined the association between LVV and delayed infarction on imaging found that 31% of patients with moderate-to-severe LVV and only 4% of patients with mild or no LVV suffered cerebral infarction, although 28% of the infarcts occurred outside the territory of vessels with moderate-to-severe LVV (11).

DCI remains a significant treatable contributor to morbidity in aSAH patients. While the pathophysiology of DCI continues to be under investigation, evaluation of vascular caliber remains important due to its simplicity and moderate strength in determining the risk of DCI. Transcranial Doppler (TCD) is most commonly used to identify developing LVV (12). However, TCD is highly operator dependent, can only evaluate the most proximal vessel segments, is prone to errors related to angle of insonation, and demonstrates inconsistent accuracy (13, 14). In addition, several patients lack acoustic windows (15). CTA is an attractive modality due to its wide availability and non-invasive nature. The major disadvantages are exposure to radiocontrast and radiation, and, as a consequence, the inability to perform daily assessment. Evaluation of CTA for determining the presence of vasospasm in comparison with the gold standard of digital subtraction angiography (DSA) has revealed high sensitivity and specificity. For central vasospasm, sensitivity and specificity are about 91–92% and 73–90%, respectively. For peripheral vasospasm, these are slightly lower, with a sensitivity and specificity of 82–90% and 50–69%, respectively (16). Our study suggests that an optimal multimodal approach may combine evaluation of vascular caliber with other modalities. Non-invasive modalities shown to predict or identify the presence of DCI include CTP (16), continuous electroencephalography with measurement of the alpha-delta ratio or percentage alpha variability (17), and monitoring of the TCD pulsatility index (18). Invasive monitoring with cerebral oximetry (19), cerebral microdialysis (20), and thermal diffusion flowmetry may also be useful for the early detection of DCI (21). Of note, most of these modalities achieve early detection of DCI, rather than identification of patients at low risk who may be triaged to a lower intensity of care.

Our study has several limitations. It is retrospective, done at a single center, and the sample size is small, although all data including test positivity (LVV on CTA) and disease positivity (occurrence of DCI) were prospectively identified and documented. Screening CTA for risk-stratification was not performed consistently and was at the discretion of the attending physician. Patients who underwent CTA are likely to have been perceived as being at higher risk for DCI by the clinical team, thereby altering the pretest probability of disease. While screening CTP was sometimes performed, the sample size of such patients was insufficient for meaningful analysis of predictive ability and diagnostic accuracy at the time of completion of this manuscript. The presence of moderate-to-severe LVV was adjudicated subjectively—CONSCIOUS-1 criteria were followed using visual estimates rather than measurement with calipers. The diagnosis of DCI, although prospective, was inherently subjective. UMSAHOD investigators adjudicating the clinical diagnosis of DCI were not blinded to the CTA results. Our focus was on the prediction of DCI, rather than long term outcomes; prior studies have addressed the association between LVV, DCI, and long-term outcomes.

In conclusion, the presence of moderate-to-severe LVV on screening CTA performed between days 4 and 8 following aSAH was an independent predictor of DCI, but achieved only moderate diagnostic accuracy, with NPV 86% and sensitivity 54%. Complementary risk-stratification strategies are likely necessary.

## Data Availability Statement

The raw data supporting the conclusions of this article will be made available by the authors, without undue reservation.

## Ethics Statement

The studies involving human participants were reviewed and approved by University of Michigan Institutional Review Board (IRBMED). Written informed consent for participation was not required for this study in accordance with the national legislation and the institutional requirements.

## Author Contributions

AN, CW, AP, KS, and VR: contributed to conception and design of the study. CW, KS, and VR: organized the database. VR: performed the statistical analysis. AN and VR: wrote the first draft of the manuscript. All authors contributed to manuscript revision, read, and approved the submitted version.

## Conflict of Interest

The authors declare that the research was conducted in the absence of any commercial or financial relationships that could be construed as a potential conflict of interest.

## Publisher's Note

All claims expressed in this article are solely those of the authors and do not necessarily represent those of their affiliated organizations, or those of the publisher, the editors and the reviewers. Any product that may be evaluated in this article, or claim that may be made by its manufacturer, is not guaranteed or endorsed by the publisher.
